# Questions of a Correspondent

**Published:** 1862-07

**Authors:** 


### QUESTIONS OF A CORRESPONDENT.

Dr. D. asks the following questions, and desires an answer to each.

1st. Would you extract a rather firm *first* cuspid tooth for a girl æt. 15 years, with a *beautiful* set of teeth inclusive? There is some evidence of the permanent tooth inside. Would taking out the temporary tooth enable the permanent tooth to come sooner or better? What is best?

2d. A child, five years old, has a central superior incisor and a first temporary molar dead, and alveolar *abscesses* from their roots. To what extent is an abscess with its secretion about a permanent germ injurious?

In reply to the first question, we would say, that the propriety of extracting such tooth would depend upon two or three circumstances. If the permanent tooth is making progress, and is coming either outward or inward, making a perceptible fullness of the gum, the temporary should be extracted. In such cases, the permanent tooth only operates to a limited extent its part, in the absorption of the root of the temporary, and thus the latter may remain comparatively firm, even till after the permanent tooth is erupted. If the temporary tooth is firm, and there is no indication of the approach of the permanent, the temporary tooth should not be extracted. Where there is a tardy growth of the osseous tissues, this condition is likely to exist.

A temporary tooth may be firm in its position, either from the attachment by the unabsorbed root, or a firm embrace between the contiguous teeth. Of course the operator should always determine in regard to that before attempting to extract.

If, then, there is an increasing fullness of the gum, occasioned by the approach of the new tooth, the temporary should be removed without respect to its apparent firmness. The permanent tooth will then come forward more rapidly, and more readily assume its proper position. There is no danger of contraction in such cases.

In answer to the second question. It is not always best to remove a temporary tooth from which there is an abscess. If the abscess occurs more than a year before the proper time for shedding of the temporary tooth, we would be disposed to retain the tooth, unless it occasions great irritation, and the discharge be of an acrid, offensive character. It would certainly be much preferable to endeavor by treatment of the abscesses, to render the teeth bearable to as near the proper time for shedding as possible. The simple secretion of pus by an alveolar abscess will not materially affect the permanent tooth, for usually the walls of the sac will prevent the pus from coming in contact with the tooth. The treatment of abscesses of the temporary teeth should, of course, be less heroic than that of the permanent, though in general character the same.

When the surrounding tissues are much involved, and the difficulty can not be easily controlled, then it is better to remove the offending tooth. In proceeding with such cases, great care should be exercised. T.

